# Use of platelet concentrates in oral surgery of patients with osteonecrosis: a scoping review

**DOI:** 10.1590/0103-6440202305254

**Published:** 2023-05-15

**Authors:** Carlos Eduardo Dutra Rufato, Mayara Colpo Prado, Renan Luiz Luft, Dionatan Zauza, Yara T. C. Silva-Sousa, Mateus Bertolini Fernandes dos Santos, Rafael Sarkis-Onofre

**Affiliations:** 1 Graduate Program in Dentistry, Atitus Education, Passo Fundo, RS, Brazil.; 2 Department of Endodontics, Faculty of Dentistry, University of Ribeirão Preto, Ribeirão Preto, Brazil.; 3 Graduate Program in Dentistry, Federal University of Pelotas, Pelotas, RS, Brazil.

**Keywords:** Platelet-Rich Fibrin, Platelet-Rich Plasma, Oral Surgical Procedures, Compromised Patients, Review

## Abstract

The objective of this study was to map, through a scoping review, the evidence available in the literature on the use of platelet concentrates in compromised patients undergoing oral surgeries. Searches were performed in electronic databases for clinical studies with compromised patients undergoing oral surgery who used platelet concentrates. Only studies published in English were included. Two independent researchers carried out the selection of studies. The study design and objective, surgical procedure and platelet concentrate used, systemic involvement, analyzed outcome, and main results were extracted. A descriptive analysis of the data was performed. Twenty-two studies met the eligibility criteria and were included. Case series was the most frequent study design among the included studies (41.0%). In terms of systemic disability, 19 studies reported patients with cancer and related to surgical treatment 16 studies reported patients underwent treatment for osteonecrosis related to the use of the drug. The most used platelet concentrate was pure platelet-rich fibrin (P-PRF). In general, most studies recommend the use of platelet concentrates. Thus, the results of this study suggest that the evidence related to the use of platelet concentrates in compromised patients when undergoing oral surgeries is still initial. Also, most studies assessed the use of platelet concentrates in patients with osteonecrosis.

## Resumo

O objetivo do estudo foi mapear, através de uma revisão de escopo, as evidências disponíveis na literatura sobre o uso de agregrantes plaquetários em pacientes comprometidos e que realizaram cirurgias odontológicas. Pesquisas foram realizadas em bases de dados por estudos clínicos com pacientes comprometidos que realizaram cirurgia odontológica e usaram agragantes plaquetários. Apenas estudos em inglês foram incluídos. Dois pesquisadores independentes realizaram a seleção dos estudos. Os seguintes dados foram extraídos: desenho do estudo, objetivo, procedimento cirúrgico, agregante plaquetário usado, envolvimento sistêmico, desfecho analisado e principais resultados. Uma análise descritiva dos dados foi realizada. Vinte e dois estudos preencheram os critérios de elegibilidade e foram incluídos. Série de casos foi o desenho de estudo mais frequente entre os estudos incluídos (41,0%). Em relação ao comprometimento sistêmico, 19 estudos reportaram pacientes com câncer e em relação ao tratamento cirúrgico, 16 estudos reportaram pacientes que realizavam tratamento para osteonecrose relacionada ao uso de medicamentos. O agregante mais utilizado foi o plasma rico em fribina (P-PRF). Em geral, maioria dos estudos recomendou o uso dos agregantes plaquetários. Assim, os resultados desse estudo sugerem que a evidência relacionada ao uso de agregantes plaquetários em pacientes comprometidos que realizam cirurgia odontológica é ainda inicial. Ainda, a maioria dos estudos avaliaram o uso de agregantes plaquetários em pacientes com osteonecrose.

## Introduction

Platelet concentrates have been used in dentistry in different oral procedures, such as tooth extraction, maxillary sinus augmentation, periodontal therapy, endodontic surgery, implant dentistry, in the treatment of oral ulcers, and patients with temporomandibular disorders ^1^. The most used and reported in the literature are platelet-rich plasma (PRP) and platelet-rich fibrin (PRF), which can be pure (i.e., P-PRP, P-PRF) or leukocyte-rich (L-PRP, L-PRF), and plasma rich in growth factors (PRGF) ^2^.

The use of platelet concentrates in dentistry mainly occurs in healthy patients, where the literature has demonstrated promising results ^1^ ^,(^ ^2^ ^3^. Many of the beneficial effects of platelet concentrates are attributed to their content of bioactive molecules, specifically growth factors, which play a vital role in the healing process within the tissues. Furthermore, they can increase osteogenesis, angiogenesis, tissue regeneration ^4^ ^,^ ^5^, and act on inflammation, cell movement, and metabolism ^6^. Platelet concentrates may also have immunomodulatory effects, inhibiting cytokine secretion, and promoting tissue healing ^7^.

Considering the excellent regeneration and healing potential of platelet concentrates due to their composition, compromised patients who need dental surgical procedures may benefit more significantly from their use ^8^. These patients are increasingly frequent in the dental office and may present tissue healing and bone regeneration problems after oral surgery, and it is a dentist's responsibility to seek methods to improve the postoperative healing process with maximum predictability ^9^.

Despite the importance of the subject and the degree of complexity that oral surgery cases in compromised patients may have, little is known about the performance of platelet concentrates in patients with systematic conditions. Thus, a scoping review seems appropriate to better understand the evidence available about that and identify knowledge gaps that could help to base further research ^10^. Considering that, the present study aimed to map the available evidence in the literature regarding the use of platelet concentrates in compromised patients who underwent oral surgery procedures.

## Materials and methods

The design of this study was based on the recommendations of Peters et al. ^2020^ ^) (^ ^10^. The study protocol is available on the Open Science Framework platform through the link https://osf.io/jsxgd/, and the final study reporting followed the PRISMA-ScR ^11^.

### Eligibility Criteria

### Types of Participants

Patients without age restriction who underwent oral surgeries such as orthognathic surgery, third molar removal, surgical treatment for osteonecrosis, maxillary sinus lift procedures, treatment of oroantral communications, alveolar crest preservation after tooth extraction, alveolar cleft reconstructions, dental implants, periodontal plastic surgery, bone graft surgeries, and apical endodontic surgeries. Patients should have some type of systemic disability such as diabetes (type 1 or 2), chronic kidney disease, heart disease, cancer, osteoporosis, or have undergone organ transplantation or use medications that may cause some systemic change that compromises dental surgery procedures. Also, during the surgical procedure, any type of platelet concentrate must have been used.

### Concept

The concept of interest is to map the evidence available in the literature on the use of platelet concentrates in patients with systemic disabilities requiring any oral surgery procedure because, in healthy patients, a series of evidence is already available in the literature.

### Context

No restrictions were applied regarding the patients' age, place of study, type of platelet concentrate used, outcome measured in the study, and date of publication of the study. However, only studies published in English were included due to funding constraints.

### Types of evidence sources

Any type of clinical follow-up, such as randomized controlled trials, observational studies (cohort and case-control), or case series, was included. However, case series with less than 5 patients included were excluded.

### Search

Searches were performed without period restrictions in electronic databases (PubMed, Scopus, and Web of Science). The search strategy was based on PubMed Mesh terms and adapted to the other databases (Box 1). In addition, references to included studies were analyzed to identify additional studies. The last search was performed on 12/13/2022.

### Screening

The studies were selected using the EndNote program (version X7; Thomson Reuters), where duplicates were removed. Initially, a pilot test was conducted to test the agreement in the selection of studies between the two reviewers involved in this phase (C.E.D.R, M.C.P). For this, the references were randomly selected using the Excel program (Microsoft Excel for Mac, Microsoft). Two researchers independently identified the articles by first analyzing the titles and abstracts for relevance and the presence of eligibility criteria. These articles were classified as "include", "exclude", or "uncertain". Then, articles classified as included and uncertain were selected for full reading and further eligibility screening by the same two reviewers independently. Discrepancies in the selection of titles/abstracts and full-text articles were resolved through a discussion. In case of disagreement, the opinion of a third reviewer was obtained R.S.O.

### Data collect

A standardized data extraction form was created using the Excel program (Microsoft Excel for Mac, Microsoft). First, ten included studies were selected to test data extraction and ensure consistency in the interpretation of items. Next, the pilot test was conducted through a discussion between the three reviewers involved in this study phase to discuss all the extracted data. Subsequently, two reviewers extracted half of the included studies each (C.E.D.R, M.C.P), and a third reviewer verified the consistency of the data R.S.O.

The following data were extracted: study design (randomized clinical trials, observational studies -cohort and case-control- or case series, or others), number of participants, study objective, systemic disability, how the disability diagnosis was performed for inclusion in the study and whether the patient was stable or not. It was also collected the surgical procedure performed (orthognathic surgery, third molar removal, treatment for osteonecrosis of the bone, sinus lift procedures, treatment of intraoral communications, alveolar crest preservation after tooth extraction, alveolar cleft reconstructions, dental implants, gingival plastic surgery, bone graft surgeries, and apical endodontic surgery), platelet concentrate used (P-PRP, L-PRP, PRGF, P-PRF, and L-PRF), analyzed outcome and main results.

**Box 1:**
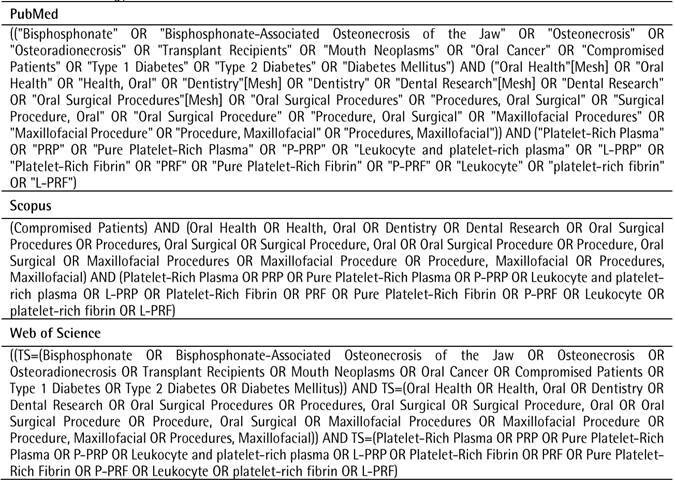
Search strategy

### Data analysis

Analyzes were performed using Stata software (version 14.0, StataCorp LLC). A descriptive analysis of the data was performed, considering the different systemic compromises, platelet concentrate used, and oral surgery procedures.

## Results

**Figure 1:**
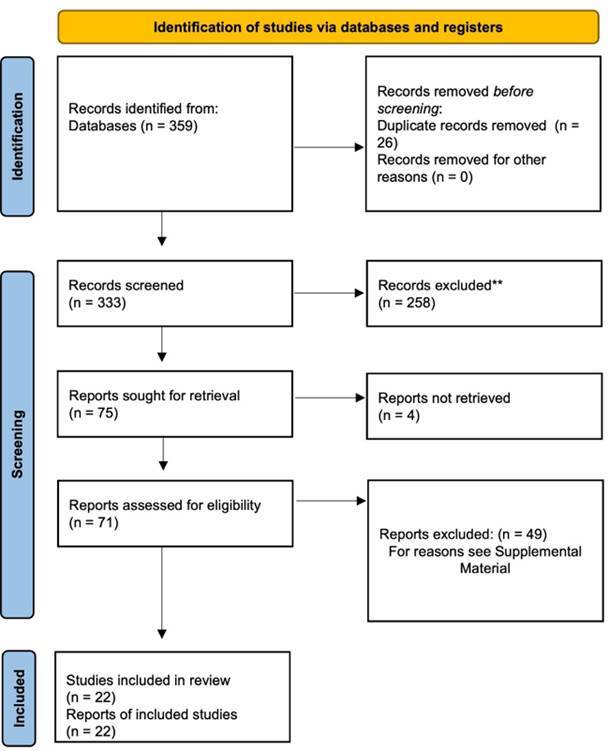
Study selection flow diagram

The search in the selected databases resulted in the identification of 359 studies. Twenty-six duplicates were removed, resulting in 333 articles. After analyzing the titles and abstracts, 258 articles were removed, resulting in 75. From there, it was not possible to obtain the full text of 4 articles, even after contacting the authors by e-mail. The 71 reports evaluated for eligibility had their full texts analyzed, and 49 of them were excluded, resulting in 22 studies included in the present scoping review. Figure 1 shows the flow diagram of the study selection. The list of excluded studies with reasons is presented through the link https://osf.io/vfxyh.

Table 1 illustrates the characteristics of the included studies. The most frequent study design was case series (n=9, 41.0%). In terms of systemic disability, 19 studies (57.6%) reported patients with cancer. Consequently, most studies (72.7%) treated osteonecrosis related to the use of a drug as a surgical procedure. The most cited platelet concentrate was P-PRF in 8 studies (36.4%), followed by L-PRF in 6 (27.3%). Twelve studies (54.5%) did not report if the patients in their research were controlled/stable during the study. Most studies (n=10, 45.5%) did not report how the diagnosis of disability or control was performed for the inclusion or exclusion of patients in the study. None of the 22 studies included reported the patient laboratory test values to determine inclusion or exclusion in the study.

**Table 1:** Characteristics of the included studies

Characteristic	N (%)
Study design	
Case series	9 (41.0)
Randomized controlled trial	4 (18.2)
Retrospective clinical study	3 (13.7)
Prospective cohort study	2 (9.1)
Case-control	1 (4.5)
Retrospective cohort study	1 (4.5)
Prospective study	1 (4.5)
Undefined#	1 (4.5)
Systemic commitment*	
Cancer	19 (57.6)
Osteoporosis	11 (33.3)
Rheumatoid arthritis	2 (6.1)
Paget's disease of bone	1 (3.0)
Surgical procedure	
Treatment of osteonecrosis related to the use of bisphosphonates	16 (72.7)
Tooth extraction	6 (27.3)
Platelet concentrate	
P-PRF	8 (36.4)
L-PRF	6 (27.3)
P-PRP	5 (22.7)
PRGF	3 (13.6)
Was the patient-controlled/stable during the study?	
Not reported	12 (54.5)
Yes	6 (27.3)
No	4 (18.2)
How was the diagnosis of impairment or control made for the inclusion or exclusion of patients from the study?		
Not reported	10 (45.5)
Not clear	7 (31.8)
Medical history	3 (13.6)
Self-report	2 (9.1)
Did the study present values of laboratory tests of the patients to determine the inclusion or exclusion of the study?		
No	22 (100.0)

# One study was classified as “Undefined” because the study design reported by the authors featured a retrospective and a prospective comparison group

* More than one impairment may have been observed in different patients in a single study

Box 2presents the analyzed outcomes and the results of the included studies. Six studies evaluated the prevention or treatment of drug-related osteoradionecrosis and osteonecrosis of the jaw using L-PRF as a platelet concentrate ^12^ ^,^ ^13^ ^,^ ^14^ ^,^ ^15^ ^,^ ^16^ ^,^ ^17^. One of them is a randomized clinical trial by Palma et al. ^12^, in which platelet concentrates did not offer additional benefits compared to the benefits achieved only with the surgical and drug protocol used in extractions in patients with post-irradiated head and neck cancer to prevent osteoradionecrosis. In the other five studies ^13^ ^,^ ^14^ ^,^ ^15^ ^,^ ^16^ ^,^ ^17^, the results showed a positive effect in the use of platelet concentrate as an adjuvant to other procedures for patients who required antiresorptive therapy and had complications of osteonecrosis of the jaws. However, at least one of the studies showed the need for more clinical trials would bring significant results ^17^.

Eight studies used P-PRF as a platelet concentrate. Five studies evaluated the treatment for osteonecrosis of the mandible associated with bisphosphonates ^18^ ^,^ ^19^ ^,^ ^20^ ^,^ ^21^ ^,^ ^22^, and two studies reported that it is not possible to prove the improvement in cases of osteonecrosis of the mandible with the use of this aggregating agent ^21^ ^,^ ^22^. One of these studies, a randomized clinical trial, reported that it was not possible to establish the advantage of using P-PRF despite observing a short-term improvement in quality of life and a reduction in postoperative pain and infections ^21^. However, a prospective observational study did not show significant improvement in terms of downstaging, pain sensation, and quality of life-related to oral health ^22^. In three studies, the P-PRF was evaluated and indicated as a step in the prevention of osteonecrosis of the jaws related to the use of antiresorptive and antiangiogenic drugs in patients requiring extractions ^23^ ^,^ ^24^ ^,^ ^25^.

P-PRP was used as a platelet concentrate in five studies, and all evaluated the treatment of bisphosphonate-related osteonecrosis of the jaws. In one case series, the results were inconclusive and suggested that more studies should be carried out, despite mentioning the benefits of the concentrates ^26^. In other studies that also addressed P-PRP, the results showed improvement in tissue healing ^27^ ^,^ ^28^ ^,^ ^29^ ^,^ ^30^.

**Box 2:**
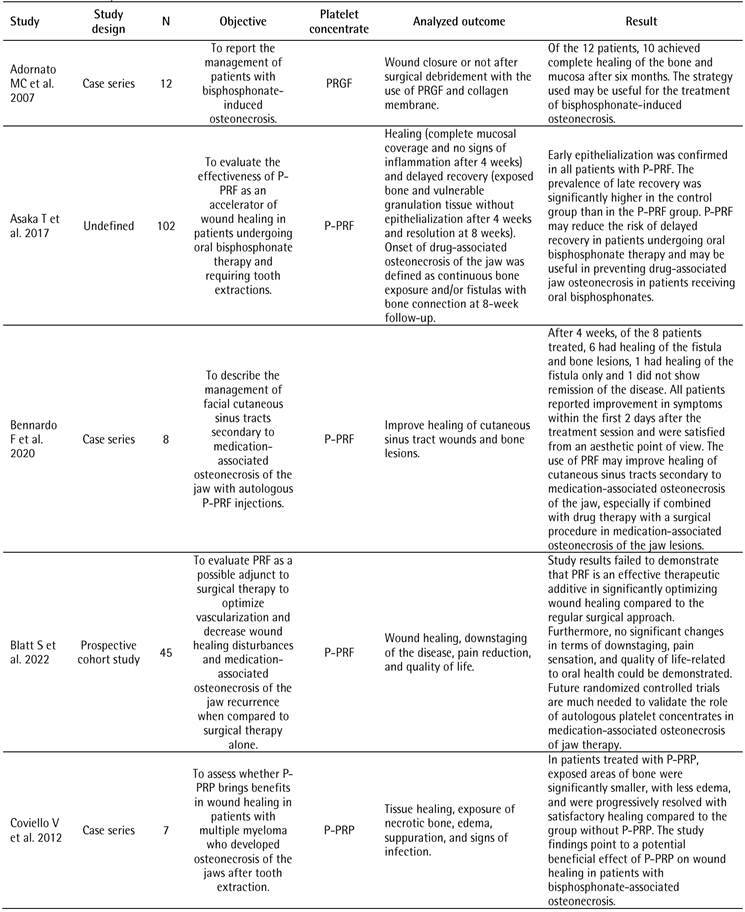
Outcomes analyzed and results of included studies.

**Box 2:**
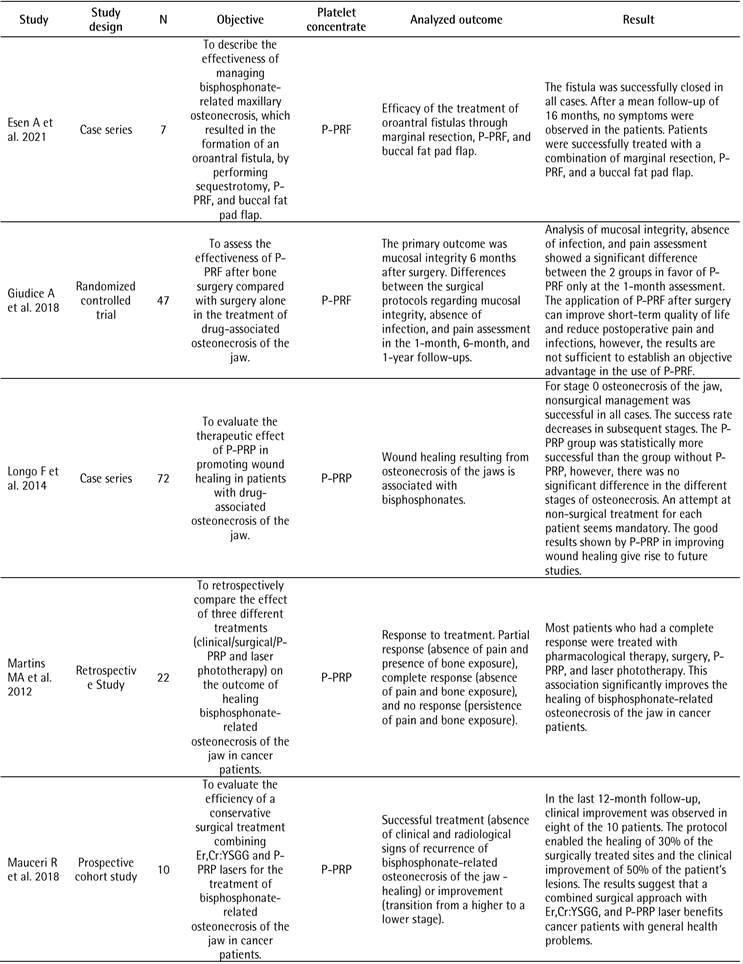
continuation

**Box 2:**
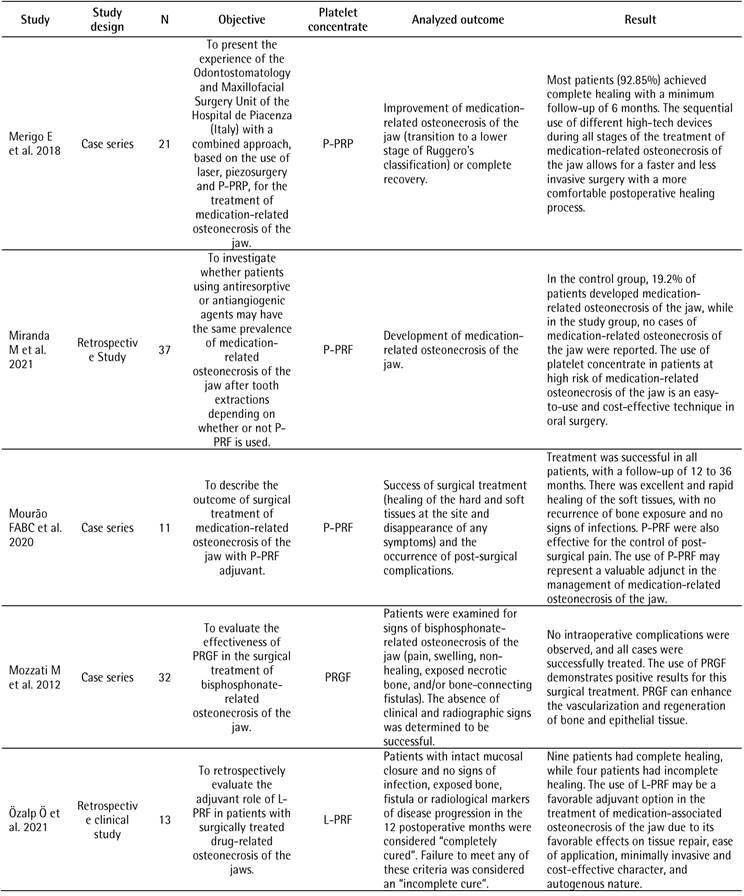
continuation

**Box 2:**
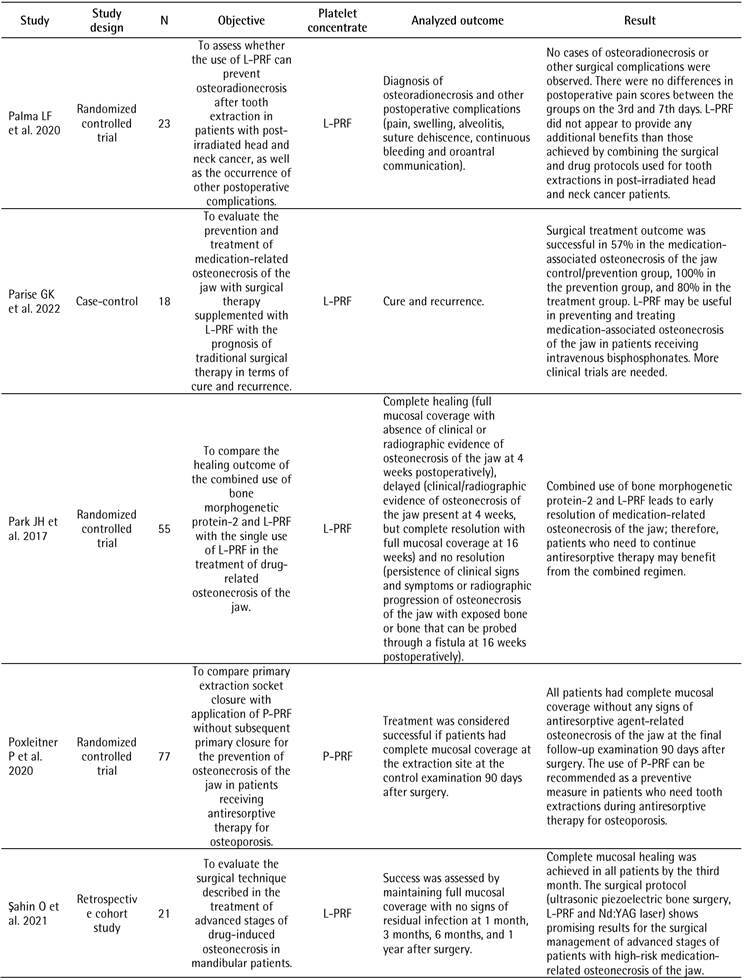
continuation

**Box 2:**
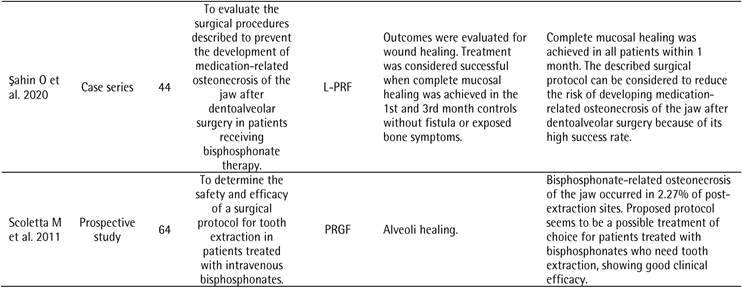
continuation

Finally, three studies used PRGF as a platelet concentrate ^31^ ^,^ ^32^ ^,^ ^33^. Two case series used the concentrates to treat osteonecrosis of the jaws related to the use of bisphosphonates and reported good results in the healing of intraoral wounds ^31^ ^,^ ^32^. Scoletta et al. ^33^ used the concentrates to determine the safety and efficacy of a surgical protocol for extracting teeth in patients treated with intravenous bisphosphonates and found that only 2.27% of cases of osteonecrosis of the jaw after the procedure.

## Discussion

This is the first study to map the evidence about the use of platelet concentrates in patients with systemic conditions who underwent oral surgery procedures. The main result identified that most of the included studies focus on patients with cancer who used bisphosphonates and, consequently, need treatment for osteonecrosis associated with the use of the such drugs. They also seem to demonstrate that platelet concentrates work well in the treatment and prevention of osteonecrosis of the jaws ^13^ ^,^ ^14^ ^,^ ^15^ ^,15,^ ^17^ ^,^ ^18^ ^,^ ^19^ ^,^ ^20^ ^,^ ^23^ ^,^ ^24^ ^,^ ^25^ ^,^ ^27^ ^,^ ^26^ ^,^ ^27^ ^,^ ^28^ ^,^ ^29^ ^,^ ^30^ ^,^ ^31^ ^,^ ^32^ ^,^ ^33^.

However, it is important to highlight that most of the evidence that supports this conclusion comes from case series located at the base of the evidence pyramid, making the results very initial, which can be classified as weak evidence ^34^. This fact is crucial considering the advancement and strengthening of evidence-based dentistry as a path to be followed by dentists who want to make clinical decisions based on scientific evidence. It is still important to point out that, although they are located at the base of the evidence pyramid, case series studies are important for building knowledge and developing hypotheses that will be tested in randomized clinical trials ^35^.

Randomized controlled trials are considered the gold standard for testing health interventions ^36^. This review included four randomized clinical trials. One of them tested P-PRF ^23^ for treating drug-associated osteonecrosis of the jaw, concluding that the local application of the concentrate can improve healing, reduce postoperative pain, and short-term infection; however, it did not demonstrate sufficient results to establish an objective advantage in the use of P-PRF. The other study with the same concentrates evaluated the prevention of osteonecrosis of the jaw associated with bisphosphonates and recommended the use of P-PRF as a preventive measure in patients with this condition who require an extraction ^25^.

 The other two randomized controlled trials analyzed tested the use of L-PRF. Palma et al. ^12^ tested L-PRF to prevent osteoradionecrosis after tooth extraction in patients with post-irradiated head and neck cancer and concluded that platelet concentrate did not provide any additional benefit compared to the benefits achieved with the combination alone of surgical and drug protocols. Park et al. ^13^ used L-PRF to treat medication-associated osteonecrosis of the jaw and showed that the combined use of platelet concentrates with morphogenetic protein-2 leads to a more satisfactory early resolution of mandibular osteonecrosis in patients who need to continue therapy with antiresorptive drugs compared to the use of the concentrates alone. Thus, even considering the four randomized controlled trials included, the evidence related to the use of platelet concentrates in patients with systemic disabilities is still initial.

Few systematic reviews on this topic were published ^37^ ^,^ ^38^. Del Fabbro et al. ^37^ addressed the use of platelet concentrates in the treatment and prevention of osteonecrosis of the jaw associated with bisphosphonates and, despite suggesting that their results should be analyzed with caution due to the low level of evidence of the included studies, the meta-analysis showed that the use of platelet concentrates as an adjunct to oral surgery procedures may have a beneficial effect. Serrano et al. ^38^ assessed whether the use of autologous platelet concentrates immediately after tooth extraction would prevent osteoradionecrosis in patients treated with radiotherapy for head and neck cancer, and according to the evidence found, a reliable statement could not be made despite studies suggesting that the use of autologous platelet concentrates does not seem to be beneficial for the evaluated cases.

The systematic reviews on the subject, and the identification of only four randomized clinical trials in our study, reinforce that the evidence on the use of platelet concentrates in patients with a systemic impairment who underwent oral surgery procedures is still very initial. The lack of sufficient data in the literature and the lack of evidence may arise from the fact that uncontrolled patients present a complicating factor for performing surgical procedures, and in many cases involving these patients, surgeries are not even indicated.

Some study limitations should be mentioned. The search was performed only in three databases and limited to studies in English, which may have limited the identification of studies. Related to the articles included, heterogeneity was identified considering the study designs and the wide range of concentrates used in different surgical procedures, making it difficult to compare them and reach a consensus on the results and future trends.

Last, it is suggested that randomized clinical trials comparing two or more platelet concentrates in the same surgical procedure be performed. Most of the included studies considered patients who used bisphosphonates and had osteonecrosis of the jaws associated with medication, demonstrating that there is also a lack of studies addressing other types of systemic involvement. As already mentioned in this discussion, studies with this population are very scarce, mainly due to the risks that surgical procedures can cause to the patient. Thus, further studies considering patients with different disabilities are needed, and consequently, the evidence can emerge and guide clinical management regarding the use of aggregators in this population. In addition, it is important to analyze the cost-effectiveness of these procedures discussed in this review since the use of platelet concentrates needs the use of equipment that, in most cases, is not present in the dentist's clinical routine.

In conclusion, the results of this study suggest that the evidence related to the use of platelet concentrates in compromised patients when undergoing oral surgery procedures is still initial Also, most studies assessed the use of platelet concentrates in patients with osteonecrosis. Lastly, it is recommended that adequate randomized clinical trials and studies that address other systemic compromises be performed to improve the level of evidence.
